# Responders and nonresponders to topical capsaicin display distinct temporal summation of pain profiles

**DOI:** 10.1097/PR9.0000000000001071

**Published:** 2023-04-04

**Authors:** Felyx Wong, Aditi Reddy, Yeanuk Rho, Jan Vollert, Paul H. Strutton, Sam W. Hughes

**Affiliations:** a The Nick Davey Laboratory, Department of Surgery and Cancer, Faculty of Medicine, Imperial College London, London, United Kingdom; b Pain Research, Department of Surgery and Cancer, Faculty of Medicine, Imperial College London, London, United Kingdom; c The Pain Modulation Laboratory, Brain Research and Imaging Centre (BRIC), School of Psychology, Faculty of Health, University of Plymouth, Plymouth, United Kingdom

**Keywords:** Capsaicin, Temporal summation, Facilitation, Responder

## Abstract

**Introduction:**

Topical application of capsaicin can produce an ongoing pain state in healthy participants. However, approximately one-third report no pain response (ie, nonresponders), and the reasons for this are poorly understood.

**Objectives:**

In this study, we investigated temporal summation of pain (TSP) profiles, pain ratings and secondary hyperalgesia responses in responders and nonresponders to 1% topical capsaicin cream.

**Methods:**

Assessments were made at baseline and then during an early (ie, 15 minutes) and late (ie, 45 minutes) time points post-capsaicin in 37 healthy participants.

**Results:**

Participants reporting a visual analogue scale (VAS) rating of >50 were defined as responders (n = 24) and those with <50 VAS rating were defined as nonresponders (n = 13). There was a facilitation of TSP during the transition from an early to the late time point post-capsaicin (P<0.001) and the development of secondary hyperalgesia (P<0.05) in the responder group. Nonresponders showed no changes in TSP or secondary hyperalgesia during the early and late time points. There was an association between baseline TSP scores and the later development of a responder or nonresponder phenotype (r = 0.36; P = 0.03). Receiver operating characteristic analysis revealed that baseline TSP works as a good response predictor at an individual level (area under the curve = 0.75).

**Conclusion:**

These data suggest that responders and nonresponders have different facilitatory pain mechanisms. The assessment of TSP may help to identify participants with stronger endogenous pain facilitation who may be more likely to respond to topical capsaicin.

## Abstract 2

Responders and nonresponders to topical capsaicin have different central facilitatory pain mechanisms.

## 1. Introduction

Topical application of capsaicin can result in the development of hyperalgesia and an ongoing pain state in healthy participants.^14^ However, its use in experimental or interventional studies is often limited because of variability in capsaicin sensitivity and the development of nonresponder phenotypes.^2,20,29^ Nonresponders are typically characterised as those experiencing a lack of ongoing pain, hyperalgesia, or allodynia and can occur in up to 40% of healthy participants.^11,17,19^ The mechanisms that underpin these high rates of nonresponders in the capsaicin model are poorly understood.

Capsaicin evokes an ongoing afferent drive to the spinal cord and the perception of ongoing pain via afferent projections to pain-related brain regions.^15,32^ The resulting phenotype often also displays patterns of hyperalgesia typically seen in neuropathic pain.^21^ As well as mimicking key symptoms of persistent pain states, the capsaicin model is also thought to affect dynamic pain processing in the brain and brainstem.^7,18,32^

Temporal summation of pain (TSP) is a psychophysical measure of endogenous pain facilitation and is typically enhanced during sensitised pain states.^27^ Temporal summation of pain is predicted to be underpinned by, among other mechanisms, the enhanced activity of spinal wide-dynamic-range neurons. These are the same neurons that underpin wind-up as measured in rodents, and therefore act as a proxy of enhanced nociceptive facilitation in the spinal cord.^1^ Facilitated TSP responses have helped to understand the central pain mechanisms that underpin pain and pain-free states in patients with chronic pain and to help predict long-term pain outcomes after surgery.^24,28^ These facilitated pain mechanisms are thought to be a key driver for the development of sensitised pain states.

Recent studies have demonstrated the benefit of measuring TSP at multiple time points to track temporal changes in sensitisation and endogenous analgesic processes within human surrogate pain models.^7,23^ Responders to topical capsaicin typically develop an ongoing pain state after approximately 45 minutes,^8^ which provides key mechanistic windows through which we can compare TSP profiles during pain and pain-free states. Critically, this also allows us to compare facilitated pain mechanisms in both responders and nonresponders to topical capsaicin, whereby we hypothesise enhanced TSP measures in conjunction with the development of an ongoing pain state. In line with this, we also anticipated that participants with higher baseline TSP scores (ie, stronger endogenous pain facilitation mechanisms in the absence of capsaicin) would be more likely to develop a responder phenotype.

## 2. Methods

### 2.1. Participant screening

All procedures were approved by the Imperial College London Research Ethics Committee (ICREC). The participants were informed of the experimental protocols and subsequently provided written consent in accordance with the principles of the Declaration of Helsinki. In this study, 37 healthy participants (mean age [SD] = 20.1 ± 2.9 years; 18 female participants) were recruited from Imperial College London and were initially screened to see if they met any of the exclusion criteria for pain testing (ie, pregnancy, diabetes, blood disorders, neurological conditions, immune suppression, inflammatory disease, psychiatric conditions, and taking steroid, antibiotic, or pain medicines).

### 2.2. Response to topical capsaicin

All participants received topical application of capsaicin cream (1% wt/wt; Pharmacierge, London, United Kingdom). Using a 1-mL syringe, 50 µL was ejected onto a 9-mm-diameter clear plastic disc that was then placed face-down on an area of the L5 dermatome one-third the way along a line from the left fibula head to the left lateral malleolus (area of capsaicin skin contact = 64 mm^2^). The participants used a modified visual analogue scale (VAS) used previously,^6,8,10,11^ where 0 = no sensation; <50 = nonpainful sensation; >50 = ongoing pain; and 100 = worst pain imaginable. After application of capsaicin cream, the participants were instructed to rate the sensation every 3 minutes for 60 minutes. The participants described the sensation initially as a nonpainful “tingling” (ie, <50 VAS rating) that increased in intensity over approximately 45 minutes until a distinct ongoing “stinging” or “burning” pain was perceived (ie, >50 VAS rating). We defined a responder phenotype to capsaicin as having a sustained pain elicited by capsaicin calculated over a 15-minute period between 45 and 60 minutes after capsaicin application (ie, VAS rating above 50 in the late time point^11^). Using 50 VAS rating threshold, we found there to be a 35% nonresponder rate (ie, participants who did not experience any ongoing pain >50 VAS rating throughout the study period), leaving 24 responders (mean age [SD] = 21.3 ± 3.2 years; 9 female participants) and 13 nonresponders (mean age [SD] = 20.3 ± 1.4 years; 9 female participants).

### 2.3. Temporal summation of pain testing

**Figure 1.**
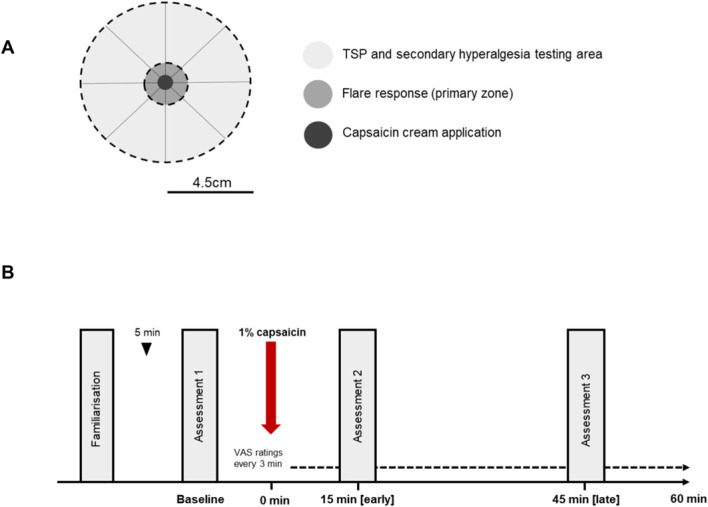
Experimental protocol. (A) Schematic showing sites of topical capsaicin cream application alongside primary and secondary testing zones. (B) All participants were first familiarised with all testing procedures before baseline responses to single and repeated 256 mN pin prick stimuli were determined. Topical capsaicin (1%, 50 µL) was then ejected onto a 9-mm-diameter clear plastic disc that was placed face-down on the L5 dermatome. Visual analogue scale (VAS) ratings were then recorded every 3 minutes throughout the rest of the protocol. At 15 (ie, early pain-free period) and 45 (ie, ongoing pain period) minutes post-capsaicin application, responses to single and repeated 256-mN pin prick stimuli were remeasured. TSP, temporal summation of pain.

Temporal summation of pain was determined at one point in an area adjacent to the capsaicin cream application using a mechanical pinprick stimulator (256 mN; 0.25 mm tip diameter; MRC Systems GmbH, Heidelberg, Germany; Fig. 1A). A standard VAS (range: 0 = no pain, 100 = worst pain imaginable) was used to determine the pain intensity at the end of a single stimulus. Ten seconds later, a series of 10 successive stimuli were applied with a frequency of 1 Hz within the same skin area of ∼1 cm^2^. A combined assessment of pain intensity at the end of the series of 10 stimuli was also determined by taking VAS ratings, which was compared with the pain intensity rating given after a single stimulus. This procedure was repeated 5 times.^30^ Temporal summation of pain was then calculated as the absolute change in VAS rating between the single stimulus and the repeated stimuli, which reflects the changes in pain perception across the series.^24,26^

### 2.4. Secondary hyperalgesia testing

Secondary mechanical hyperalgesia was assessed using a 256-mN pin prick stimulator (0.25 mm tip diameter; MRC Systems GmbH), whereby mean pain ratings to 5 consecutive stimuli were collected before capsaicin and at 45 minutes after capsaicin. Pre- and post-capsaicin pain ratings were then log-transformed to produce a normal distribution.^30^

### 2.5. Experimental protocol

All participants attended a single session at room temperature and were seated on a couch with knees extended at 180° and hip flexed at 90°. First, participants were familiarised with the use of a 256-mN pin prick stimulator in line with the German Research Network on Neuropathic Pain (DFNS) guidelines.^30^ Next, baseline pain ratings to single and repeated pin prick stimuli were determined before topical application of 50 μL of 1% capsaicin cream over the skin on the L5 dermatome (covering an area of 64 mm^2^). All measurements post-capsaicin were made while the capsaicin cream was still in place on the skin. Visual analogue scale ratings were then recorded every 3 minutes to track the development of an ongoing pain state. At 15 minutes post-capsaicin application (ie, during the early time point), TSP responses were measured after the most recent ongoing pain VAS rating. At 45 minutes post-capsaicin application (ie, during the late time point), secondary hyperalgesia and TSP responses were remeasured (Fig. 1B).

### 2.6. Statistical analysis

**Figure 2.**
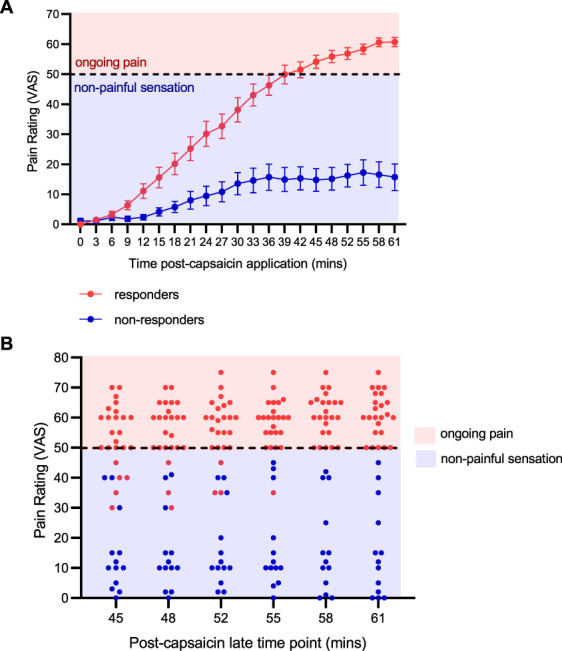
Development of responder and nonresponder profiles. (A) Changes in ongoing VAS ratings in responders and nonresponders after capsaicin application. Data are expressed as mean ± SEM. (B) Individual data for responders and nonresponders during the late time point post-capsaicin application. Red shaded area = pain; blue shaded area = nonpainful sensation. Red symbols represent responders and blue symbols represent nonresponders. *N* = 24 responders; *N* = 13 nonresponders. *P < 0.05. VAS, visual analogue scale.

All data were analysed for normality, and statistical significance was set at *P* < 0.05. The early time point was defined as the mean 15-minute period that produced a pain-free VAS rating (ie, <50 VAS score; between 15 and 30 minutes post-capsaicin application). The later time point was defined as the mean 15-minute period that produced stable ongoing pain state (ie, >50 VAS score; between 45 and 60 minutes post-capsaicin application) in the responders (Fig. 2).

Individual pain ratings to single pin prick stimuli (ie, assessment of secondary hyperalgesia) were z-transformed. Intraindividual z-score comparisons relative to the same area before capsaicin were made using the formula:

$$\text{z}-\text{score}=\frac{\text{single}\;{\text{value}}_{\text{post}-\text{capsaicin}}-\text{arithmetic}\;{\text{mean}}_{\text{pre}-\text{capsaicin}}}{\text{standard}\;{\text{deviation}}_{\text{pre}-\text{capsaicin}}}$$

The Z-scores indicate the extent to which capsaicin causes pain ratings to deviate from the pre-capsaicin (ie, baseline) distribution. Z-scores above zero indicate a gain in function (ie, more sensitive), and z-scores below zero indicate a loss of function (ie, less sensitive). Significant capsaicin-induced gain or loss of function in mechanical sensitivity was analysed using a z-test, whereby the mean values were compared with the expected values of an ideal healthy population with a mean Z-score of 0 and an SD of 1.

One-way repeated-measure analysis of variance (ANOVA) was conducted for both the responder and the nonresponder TSP data (within-subject factor = time: pre-capsaicin, early, late) with Bonferroni multiple comparison post hoc analysis. An unpaired *t* test was used to compare baseline TSP scores between the responder and nonresponder groups. A point-biserial correlation was used to correlate baseline TSP ratings and the presence or absence of responder phenotype. The relationship between baseline TSP and TSP in the late time point in responders to capsaicin was assessed using a Pearson correlation coefficient.

Receiver operating characteristic (ROC) curves, plotting sensitivity over inverse specificity, were used to estimate the ability of TSP to correctly distinguish between responders and nonresponders to topical capsaicin. The sensitivity and specificity were first calculated for each of the 37 points of the TSP scores. The sensitivity (ie, rate of correctly detected responders) were then plotted against 1 − specificity (also called false-positive rate) to obtain the ROC curve. The area under the curve (AUC) and its confidence intervals were then used to quantify if TSP predicts the response to capsaicin on an individual level above random level (ie, AUC and its confidence intervals not crossing 0.5). A statistically optimal cut-off point was determined as having the highest sum of the sensitivity and 1 − specificity (Youden J-index), balancing highest relationship of specificity and sensitivity.

## 3. Results

### 3.1. Responders to topical capsaicin display sustained ongoing pain ratings and the development of secondary hyperalgesia

**Figure 3.**
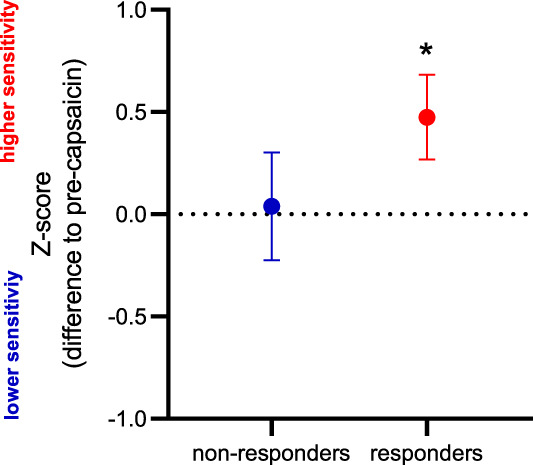
Development of secondary mechanical hyperalgesia in responders to topical capsaicin. Capsaicin-induced pain ratings in response to 256-mN pin prick stimulation in area adjacent to the flare response expressed as z-scores. Positive z-scores indicate a gain of function (ie, more sensitive), and negative z-scores indicate a loss of function (ie, less sensitive). N = 24 responders; N = 13 nonresponders. *P < 0.05.

Topical application of capsaicin led to the clear development of responders and nonresponders based on the presence of an ongoing pain state (ie, >50 VAS rating) within the 60-minute experimental window (Fig. 2A, B). We found that more female participants developed a nonresponder profile (69.2% of participants compared with 37.5% in the responder group). Responders to capsaicin were also associated with the development of mechanical secondary hyperalgesia (z-score: 0.47 ± 1.01; *P* = 0.02; d = 0.5; Fig. 3), which was not evident in nonresponders (z-score: 0.03 ± 0.95; *P* = 0.68; d = 0.1).

### 3.2. Responders show a gradual facilitation of temporal summation of pain during the development of an ongoing pain state

**Figure 4.**
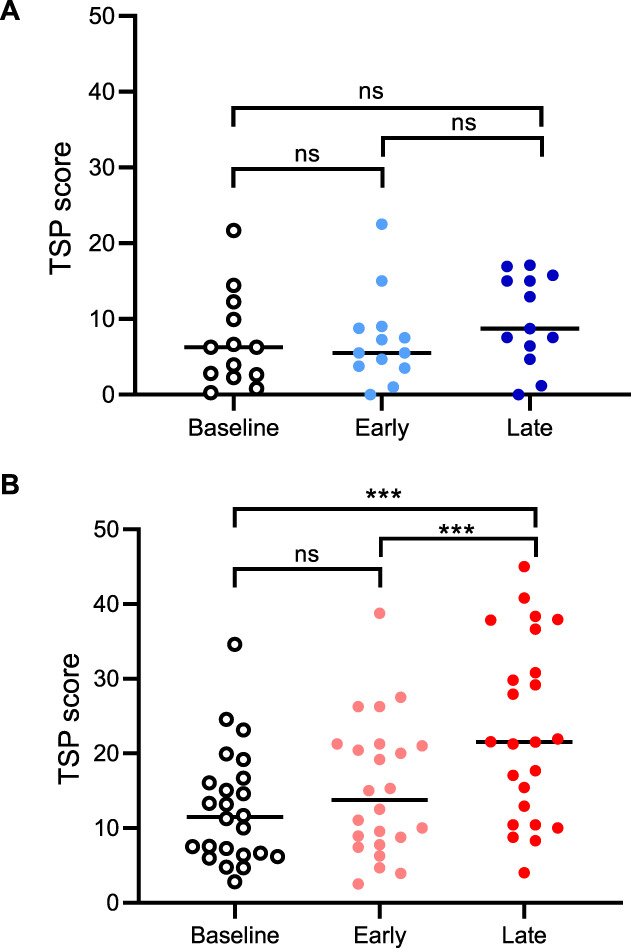
Changes in TSP in responders and nonresponders to topical capsaicin. TSP changes over time for (A) responders and (B) nonresponders. Early time point = 15 minutes postcapsaicin and late time point = 45 minutes postcapsaicin. N = 24 responders; N = 13 nonresponders; data are expressed as mean with individual data points. ***P < 0.001. ns, nonsignificant; TSP, temporal summation of pain.

In the nonresponder group, there was no change in TSP between pre-capsaicin and early and late time points post-capsaicin (F_(2,24)_ = 2.8; *P* > 0.05; ${\text{η}}_{\text{p}}^{2}$ = 0.191; Fig. 4A). However, responders showed significant changes in TSP over time (F_(2,46)_ = 27.7; *P* < 0.001; ${\text{η}}_{\text{p}}^{2}$ = 0.6; Fig. 4B). Post hoc analysis revealed significant differences in TSP between pre-capsaicin and the later time point post-capsaicin (mean difference in TSP score = 10.52; 95% confidence interval = 6.86–14.17; d = 1.4; *P* < 0.001) and between early and late time points post-capsaicin (mean difference in TSP score = 7.92; 95% confidence interval = 4.26–11.57; d = 1.09; *P* < 0.001). There was no significant difference between pre-capsaicin and the early time point TSP scores (mean difference in PSQ score = 2.60; 95% confidence interval = −1.05 to 6.26; d = 0.36; *P* = 0.25).

### 3.3. Higher baseline temporal summation of pain scores is associated with the development of a responder phenotype

**Figure 5.**
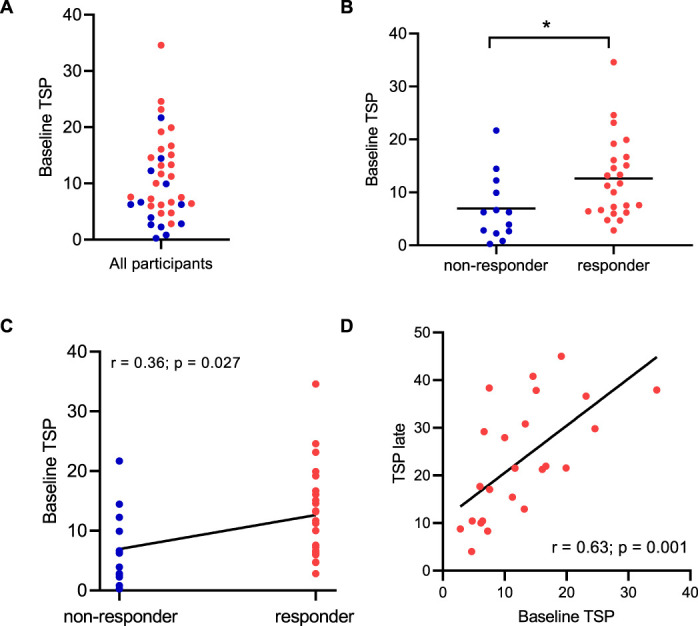
Baseline TSP profiles in responders and nonresponders. (A) Individual data points for baseline TSP scores in responders (red) and nonresponders (blue). (B) Comparison of baseline TSP scores in responders and nonresponders. (C) Point biserial correlation demonstrating an association between higher baseline TSP scores and the later development of a responder phenotype (ie, >50 VAS rating measured during the later time point). (D) Relationship between baseline TSP and TSP in the later time point in responders. N = 24 responders; N = 13 nonresponders. *P < 0.05. TSP, temporal summation of pain.

Responders and nonresponders displayed different TSP profiles at baseline (TSP scores of responders: 12.63 ± 7.64 vs nonresponders: 6.93 ± 6.19; *P* = 0.02; Fig. 5B). A point-biserial correlation was run to determine the relationship between a continuous level variable (ie, TSP scores at baseline) and a binary variable (ie, responder or nonresponder phenotype). There was a positive correlation between baseline TSP score and phenotype (*r*_pb_ = 0.363; 95% confidence interval = 0.04–0.61; *n* = 37, *P* = 0.027; Fig. 5C). There was a significant correlation between baseline TSP and TSP in the late time point in responders (*r* = 0.63; *P* = 0.001; Fig. 5D).

### 3.4. Baseline temporal summation of pain scores can predict the response to topical capsaicin

**Figure 6.**
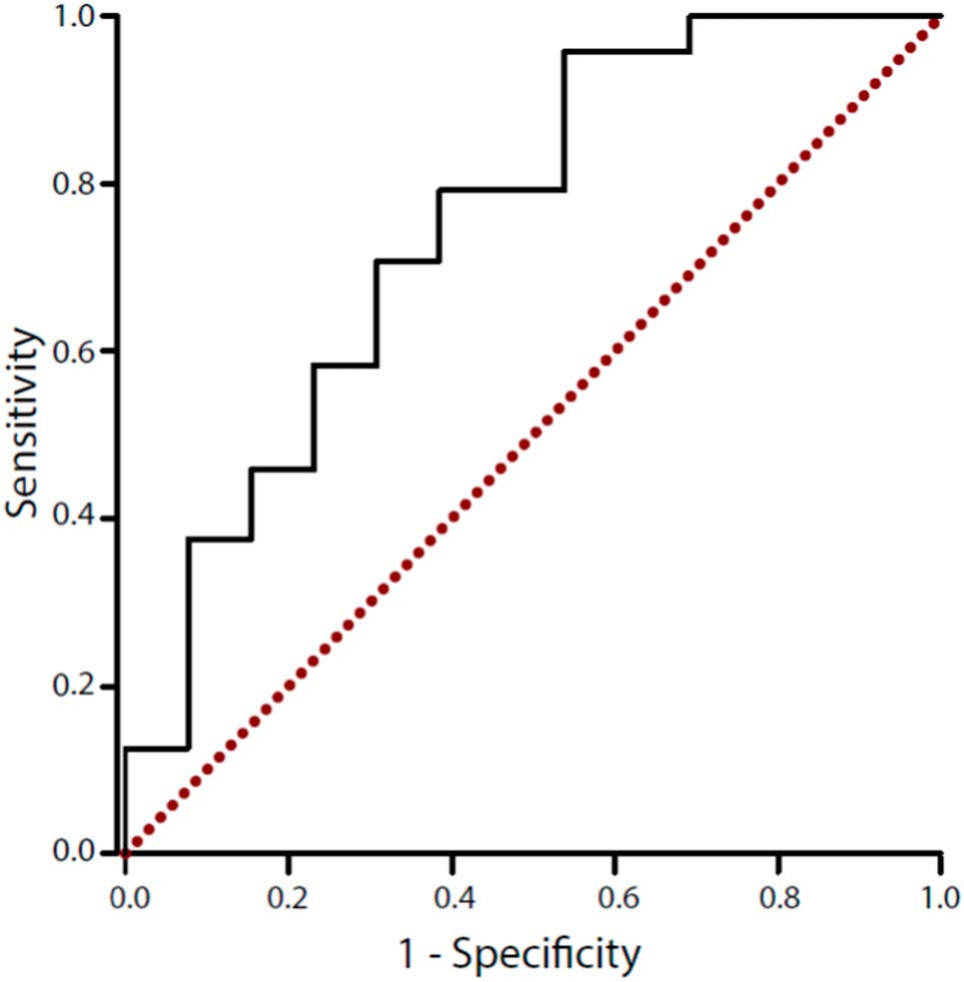
Receiver operating characteristic curve for TSP as a response predictor. The plot displays sensitivity (true positive rate) over inversed specificity (false-positive rate). Each edge represents a specific TSP cut-off. Generally, values in the upper left corner of the plot are favourable (high sensitivity with high specificity). A random classification would produce values along the dotted diagonal. TSP, temporal summation of pain.

Receiver operating characteristic analysis indicates that TSP is a good predictor for the response to topical capsaicin (AUC = 0.75; 95% confidence interval = 0.58–0.92; Fig. 6). Youden J-index indicated 3 possible cut-off values with similar high overall values: 4.3 (J: 0.42, sensitivity: 96%, specificity: 46%), 6.3 (J: 0.41, sensitivity: 79%, specificity: 62%), and 7.0 (J: 0.40, sensitivity: 71%, specificity: 69%). Although 4.3 constitutes the highest J, unless sensitivity is of highest importance, we would recommend 6.3 as cut-off because the balance between sensitivity and specificity is more even.

## 4. Discussion

In this study, we have shown that responders and nonresponders to topical capsaicin display different TSP profiles. We show that responders develop secondary mechanical hyperalgesia and a facilitated TSP response during the development of an ongoing pain state, whereas TSP in nonresponders remains unchanged over the same period. We have also demonstrated that responders and nonresponders have different baseline TSP values, and higher TSP scores measured at baseline were associated with the development of a responder phenotype. Critically, we show that baseline TSP scores also act as a good response predictor to topical capsaicin using ROC analysis. These data suggest that responders and non-responders to topical capsaicin display different facilitatory pain mechanisms in the central nervous system.

The topical capsaicin model differs to other human surrogate pain models in that it is nonpainful to induce and includes a relatively long development phase, during which VAS ratings slowly increase until an ongoing burning pain is perceived.^6,8,13,22^ In the early stages after capsaicin cream application, we found that it is difficult to distinguish between responders and nonresponders based on nonpainful VAS ratings (ie, <50 VAS ratings). Despite this, the trajectory of facilitated TSP responses appeared different between responders and nonresponders. It is possible that responders start to display further facilitated pain mechanisms at the spinal level (ie, because of gradual sensitisation of dorsal horn neurons) during an acute period after induction of the capsaicin model, which precedes the onset of an ongoing pain state and secondary hyperalgesia. The facilitated TSP responses seen between the early and late time points are likely to be a manifestation of the plasticity induced within the dorsal horn as a result of the increasing capsaicin-induced ongoing afferent drive, which was accompanied by secondary mechanical hyperalgesia.^14–16^ Neuroimaging studies have also shown capsaicin-induced activation of brainstem regions involved in the descending facilitation of pain signals in the spinal cord.^12,32^ The stronger endogenous pain facilitatory mechanisms seen in the present study could therefore be a key mechanistic feature that is associated with the gradual sensitisation to topical capsaicin seen in healthy participants.

We have demonstrated that higher TSP scores measured at baseline were associated with a stronger chance of developing a responder phenotype. It is therefore possible that the sensitivity to capsaicin could be determined by the dynamic nature of endogenous pain facilitatory mechanisms, whereby participants with higher TSP scores are more susceptible to developing a responder phenotype. The higher baseline TSP scores could be a result of cortical influences on descending pain modulation systems; day-to-day fluctuations in levels of sleep and cognitive and affective processing can exert top-down influences on spinally projecting pain modulation networks.^25,31^ It is possible that by screening participants for higher TSP scores, it may be possible to gain insight into the functional status of descending pain modulation systems, through which the likelihood of responding to topical capsaicin could be identified. These observations also mirror clinical pain conditions, where patients with higher TSP presurgery show persistent long-term pain outcomes after surgery.^3,28^ A larger TSP response indicates the presence of stronger background pain facilitatory mechanisms in the spinal cord. It is possible that the higher baseline scores seen in healthy participants increases the chance of capsaicin-induced afferent drive that will result in the development of a sensitised pain state.

It is also important to note that the participants in the current study group were young adults. Given that the activity in descending pain modulation networks diminishes with age,^5^ and that descending modulation of spinal processes is likely to mechanistically underpin TSP, it is possible that performing these assessments in an older population is likely to yield a higher proportion of responder phenotypes with higher baseline TSP. This is important when considering the potential clinical utility of TSP as a biomarker of vulnerability for developing chronic pain (eg, postsurgically), which could be dependent on age-related changes in descending modulation, and therefore, more research is required in this area.

Our study provides mechanistic insight into the changes in dynamic pain processing that occur during the development of a responder phenotype. However, 35% of healthy participants did not show any changes in TSP during the early or late phase despite undergoing the same induction procedure as those showing a responder phenotype. It has been previously demonstrated that TSP is only facilitated during painful episodes in both experimental and clinical low back pain.^23,24^ It is likely that this enhanced nociceptive facilitation is a result of fluctuating sensitisation at the level of the spinal cord as a result of local and descending influences, which could be driving higher TSP responses. Given that nonresponders to topical capsaicin display no clear signs of sensitisation to pain, it is possible that TSP responses remain at the same non-sensitised level during the post-capsaicin period.

In the present study, we did not measure temporal changes in endogenous pain inhibition; however, others have shown that conditioned pain modulation (CPM) responses slowly diminished over time in responders to capsaicin.^7^ It could therefore be speculated that in our nonresponder group there may be a stronger top-down inhibitory influence that could be working to suppress the expression of a responder phenotype. Interestingly, similar inhibitory mechanisms have been observed in preclinical models of neuropathic pain, where pharmacological blockade of descending inhibition can reveal sensitisation in previously non-neuropathic pain states.^4,9^ Future research should aim to explore the effects of capsaicin-induced ongoing afferent drive on top-down inhibitory control in responders and nonresponders.

Early studies aiming to identify the reasons for reduced sensitivity or nonresponse to topical capsaicin have shown that the response is temperature dependent and that the means by which capsaicin is applied can affect penetration through the skin.^20^ Although these methodological considerations are clearly important for capsaicin pharmacokinetics, they can be largely controlled for by monitoring the temperature of the room and ensuring an even application of capsaicin cream, which we achieved in the present study using a constant room temperature and a clear disc to optimise the even contact with the skin. Despite this, it cannot be ruled out that the response to capsaicin could, in part, be determined by small fluctuations in skin temperature between participants. However, it is apparent that a key mechanistic feature that helps to distinguish responders from nonresponders to topical capsaicin is the enhanced TSP response, indicative of a shift towards enhanced endogenous pain facilitation during the development of an ongoing pain state. It is also possible that nonresponders display different skin properties, such as thickness, lower density of afferents, or lower absorption that could have contributed to the reduced peripheral activation of C-fibre nociceptors. Future studies should aim to record electrophysiological activity from peripheral nociceptors in nonresponders to better understand the contribution of peripheral mechanisms to nonresponder profiles.

In summary, we have shown that healthy participants can show different temporal patterns of central pain facilitatory mechanisms in response to topical capsaicin. We have shown that the presence of facilitated TSP responses is a key feature of the transition into an ongoing pain state, which is accompanied by the development of secondary mechanical hyperalgesia. Future research that adopts an early assessment of TSP may help to identify healthy participants who are likely to develop a responder phenotype.

## Disclosures

The authors have no conflict of interest to declare.
